# Laser emission at 675 nm: In vitro study evidence of a promising role in skin rejuvenation

**DOI:** 10.1016/j.reth.2023.01.007

**Published:** 2023-02-14

**Authors:** Giada Magni, Laura Pieri, Irene Fusco, Francesca Madeddu, Tiziano Zingoni, Francesca Rossi

**Affiliations:** a Istituto di Fisica Applicata "Nello Carrara", Consiglio Nazionale delle Ricerche (CNR - IFAC), Via Madonna del Piano, 10, Sesto Fiorentino, Firenze, 50019, Italy; b El.En. Group, Via Baldanzese, 17, 50041, Calenzano, Italy

**Keywords:** 675-nm laser wavelength, Laser treatment, Skin aging, Skin resurfacing, Neocollagenesis, Confocal microscopy

## Abstract

Light-based therapies have been proven to influence and perhaps reverse skin ageing at clinical, molecular and histologic levels. Laser technology decreases photodamage by promoting collagen type I and III synthesis and enhancing the expression of heat shock protein. *Aims*: This study aims to assess different doses of 675 nm irradiation on human dermal fibroblast cells to evaluate the potential therapeutic effects on the rejuvenation process. *Methods:* This study employed a laser system that emits 675 nm wavelength: 260, 390, 520 and 650 J/cm^2^ doses were tested on adult human dermal fibroblast cells. Cellular viability, proliferation, and synthesis of type I and III collagen were studied. *Results:* No dose tested showed effects on cell viability and proliferation at 24 and 48 h from the irradiation. Doses of 260 and 520 J/cm^2^ causes a significant decrease in type I collagen fluorescence intensity, while 390 J/cm^2^ elicits a significant increase in type III collagen expression. *Conclusions:* Our results showed that 675 nm laser irradiation does not affect cell viability while modulating cell proliferation and collagen synthesis in human adult cultured fibroblasts in vitro. These findings suggest that 675 nm laser irradiation potentially plays a role in skin rejuvenation.

## Highlights

• Change in type I/III collagen ratio induces premature ageing.
• 675 nm irradiation can modulate cultured human dermal fibroblasts.
• 675 nm irradiation directly affects collagen synthesis stimulating neocollagenesis.
• Potential role of 675 nm in skin rejuvenation via modulating collagen synthesis.
• Light-based therapy can reduce photodamage.

## 1 Introduction

Photoaging due by ultraviolet (UV) irradiation are one of the main causes of premature skin ageing [1]. Photoaging includes several changes in the skin, such as deep wrinkles and changes in texture, due to an upregulation of matrix metalloproteinase 1 (MMP1) [2].

Principal pathogenic effects of the UV radiation are the production of reactive oxygen species (ROS) and the development of genetic alterations. Augmented levels of ROS induce an increase in inflammatory cytokines, which contributes to enhancing oxidative stress, giving rise to a vicious circle. Oxidative stress has an unfavourable effect on the skin. It induces a decrease and fragmentation of elastic fibers and collagen in the dermis, leading to hyperpigmentation, skin laxity and deep wrinkles [3].

Growth factors regulate collagen breakdown, the recruitment of new fibroblast cells, and the neoangiogenesis process [4]. Transforming Growth Factor – Beta (TGF-β) is activated during photoaging in the skin, and its synthesis is up-regulated. This event leads to an over-expression of Metalloproteinases (MMPs) and pro-inflammatory cytokines, thus collagen degradation, extracellular matrix (ECM) deterioration and formation of aberrant elastic fibers. On the other hand, physiological levels of TGF-β inhibit the proliferation of keratinocytes and stimulate dermal fibroblasts in ECM deposition. Furthermore, Orringer et al. [5], showed that the collagen fragmentation induced by MMPs could promote the new collagen biosynthesis.

Light-based treatments have been found to influence and perhaps reverse skin ageing by increasing the expression of heat shock protein and collagen types I and III. Changes in the typical collagen type I/III ratio in the face skin can induce premature ageing and wrinkles. Visible and near-infrared (NIR) light may cause a wide range of modifications in the cell transcriptome via various gene expression mechanisms, resulting in changes in differentiation, proliferation and collagen synthesis [6]. Red light (600–760 nm) and near-infrared light (780–1000 nm) can impact a variety of processes in live cells and tissues. These wavelengths promote chemical reactions, including interactions with intracellular water and respiratory oxygen, thus causing changes in intracellular calcium levels and oxidative stress. Additionally, modifications in metabolic processes lead to a variety of physiological changes, including cellular differentiation and proliferation. These metabolic processes are triggered by alteration of electrical capacitance of membrane, gene expression, alterations in metabolism and remodeling of cytoskeleton [7]. A retrospective study [8] on 15 patients who received broadband light treatment at least once a year for 5-11 years found that their post-treatment age was an average of 2 years younger than their actual age at study commencement, despite the fact that they aged a median of 9 years. However, Hogan [9] recommends a multifaceted approach to prejuvenation including neurotoxins, fillers and energy sources. The cumulative effects of combined prejuvenation procedures are probably synergic rather than additive. Neurotoxins appear to increase the longevity of dermal fillers by diminishing facial muscle movement and the efficacy of laser resurfacing by creating a less contractile skin surface; the wavelength of 675 nm has already proven to be effective from the clinical data in treatments of textures, scars, wrinkles and pigmentations [[10], [11], [12], [13], [14]].

Here, we applied a 675-nm laser wavelength on cultured fibroblasts to analyse the effects on proliferation and changes in type I/III collagen expression, to obtain in vitro evidence about the potential therapeutic effects in rejuvenation prejuvenation.

## 2 Materials and Methods

### 2.1 Device description

The RedTouch laser (Deka Mela, Florence, Italy) technology is based on a wavelength emission of 675 nm (red light) through micro thermal zone (“DOT” of 0.7 mm width) and a scanning system of 15 × 15 mm capable of producing a selective skin thermal damage with an average depth of 500 μm, getting to the dermis. The DOT pulses separated by untreated tissue (DOT spacing) induce a heating impact in this area, causing denaturation of collagen fibers and the production of new collagen [15]. To protect the epidermis from temperature increase, RedTouch includes an integrated contact skin cooling system.

### 2.2 Cell culture

Adult Human Dermal Fibroblast cells (HDFa) were purchased from Thermo Fisher Scientific (Milan, Italy). Dulbecco Modified Eagle Medium (DMEM) added with 10% of Fetal Bovine Serum (FBS), and 1% Glutamine and Streptomycin (PAN-Biotech GmbH, Aidenbach, Germany) was used. Cells were kept in a cell incubator under standard culture conditions, and the DMEM was refreshed every 48 h. Trypsin-EDTA 0.25% solution (Sigma-Aldrich, Milan, Italy) was used to detach HDFa when they reached 80% of confluence as the instruction of the manufacturer recommends.

### 2.3 Sample preparation for colorimetric assay and immunofluorescence

Colorimetric assay was performed in 96-multiwell plates (Greiner Bio-One Italia, Milan, Italy). 8 × 10^3^ cells were seeded in alternate rows and columns, to make sure they have enough space to carry out irradiation. Three untreated wells were utilized as controls in each experiment. Immunofluorescence samples were prepared in 35 mm Ibidi μ-Dish (GmbH, Martinsried, Germany). Each sample was starved at least 24 h before the experiments. The irradiation parameters were reported in Table 1.

**Table 1:** Irradiation parameters used on HDFa cells.

Fluence (J/cm^2^)	DOT Spacing (μm)	mJ @ DOT
260	500	1000
390	500	1500
520	500	2000
650	500	2500

### 2.4 Cell viability and proliferation evaluation

Cell viability was evaluated using Cell Counting Kit-8 (CCK-8) assay, while cell proliferation was analyzed with Sulforodhamine B-based (SRB) (Sigma-Aldrich, Milan, Italy). Tests were performed 24 and 48 h after irradiation. Absorbance was read by Multiskan FC Microplate Photometer equipped with SkanIt software (Thermo Fisher Scientific, Milan, Italy). Each test was carried out at least in triplicate.

### 2.5 Fluorescence quantification and immunocytochemical staining

The immunocytochemical protocol was conducted as follows: HDFa cells were fixed using a 3.6% paraformaldehyde solution. Permeabilization (10 min at room temperature) was performed using 0.3% Triton-X100 diluted in Phosphate Buffer Saline (PBST). The unspecific sites blocking was obtained using 10% of goat serum in PBST. Anti-type I collagen 1:400 and anti-type III collagen 1:200 primary antibodies were applied overnight diluted in PBST. AlexaFluor 555 and AlexaFluor 647 secondary antibodies were diluted 1:500 in PBST. AbCam (Cambridge, UK) provided all of the antibodies. Control experiments were performed using only secondary antibodies to exclude non-specific binding. To stain cell nuclei and mount the coverslip, fluoroshield mounting medium with DAPI was utilized. All the reagents were purchased from Sigma-Aldrich (Milan, Italy). For each sample, at least 10 random images were acquired by SP8 laser scanning confocal microscope (Leica Microsystems, Mannheim, Germany) using a 20× dry objective (NA 0.4). The fluorescence intensity signal was analyzed with ImageJ [16] as previously described [17].

### 2.6 Statistical analysis

Data obtained from CCK-8 and SRB assays were expressed as mean ± SD. Kruskal-Wallis followed by Dunn's multiple comparisons tests were performed. Immunofluorescence intensity analysis was performed using the Mann-Whitney test, and data were expressed as mean ± SEM, n = 20. GraphPad Prism 8 (San Diego, CA, USA) was used. *∗p* < 0.05 was considered statistically significant.

## 3 Results

### 3.1 Analyses of cytotoxicity and cell proliferation following cell irradiation

**Fig. 1:**
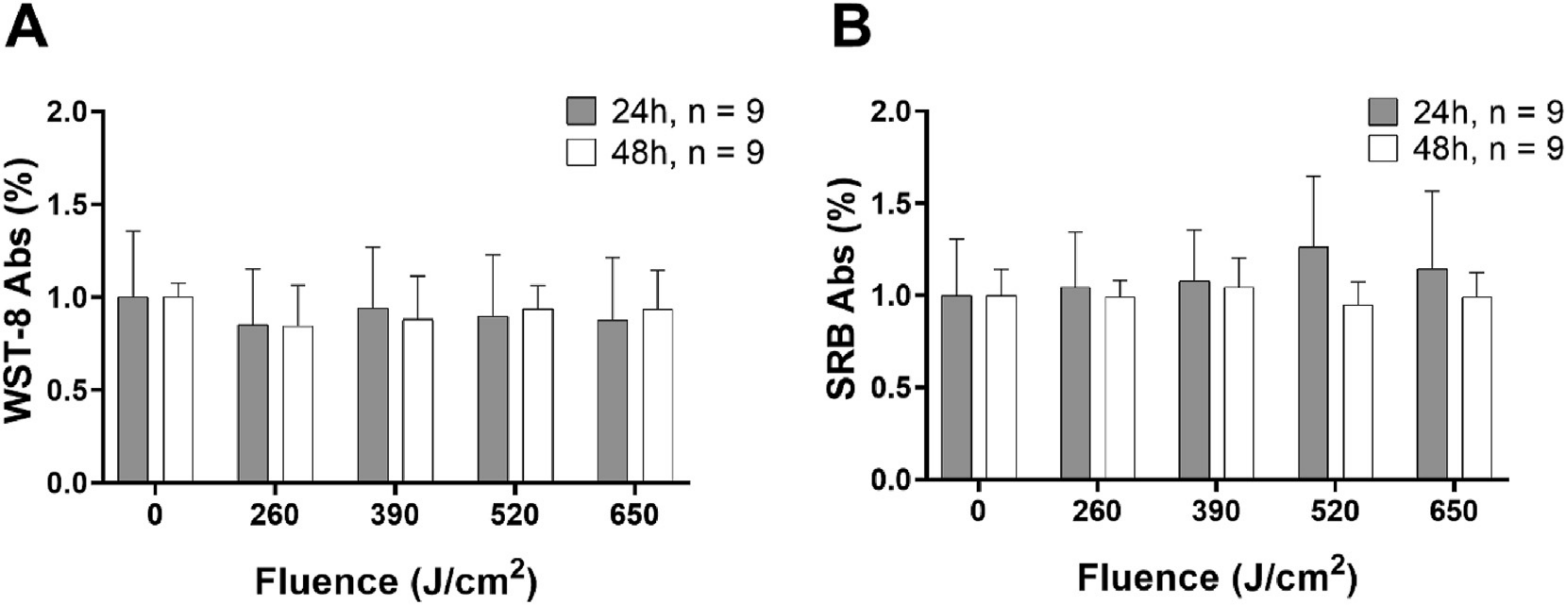
Cell viability (A) and proliferation (B) after 24 h (grey) and 48 h (white) from the irradiation. Data are expressed as mean ± SD, n = 9. Statistical analysis: Kruskal-Wallis test followed by Dunn's multiple comparisons.

CCK-8 (Fig. 1A) and SRB (Fig. 1B) tests performed on HDFa cells show that all the applied fluences did not affect cell viability and proliferation at 24 and 48 h post-treatment.

### 3.2 Semi-quantitative analysis

**Fig. 2:**
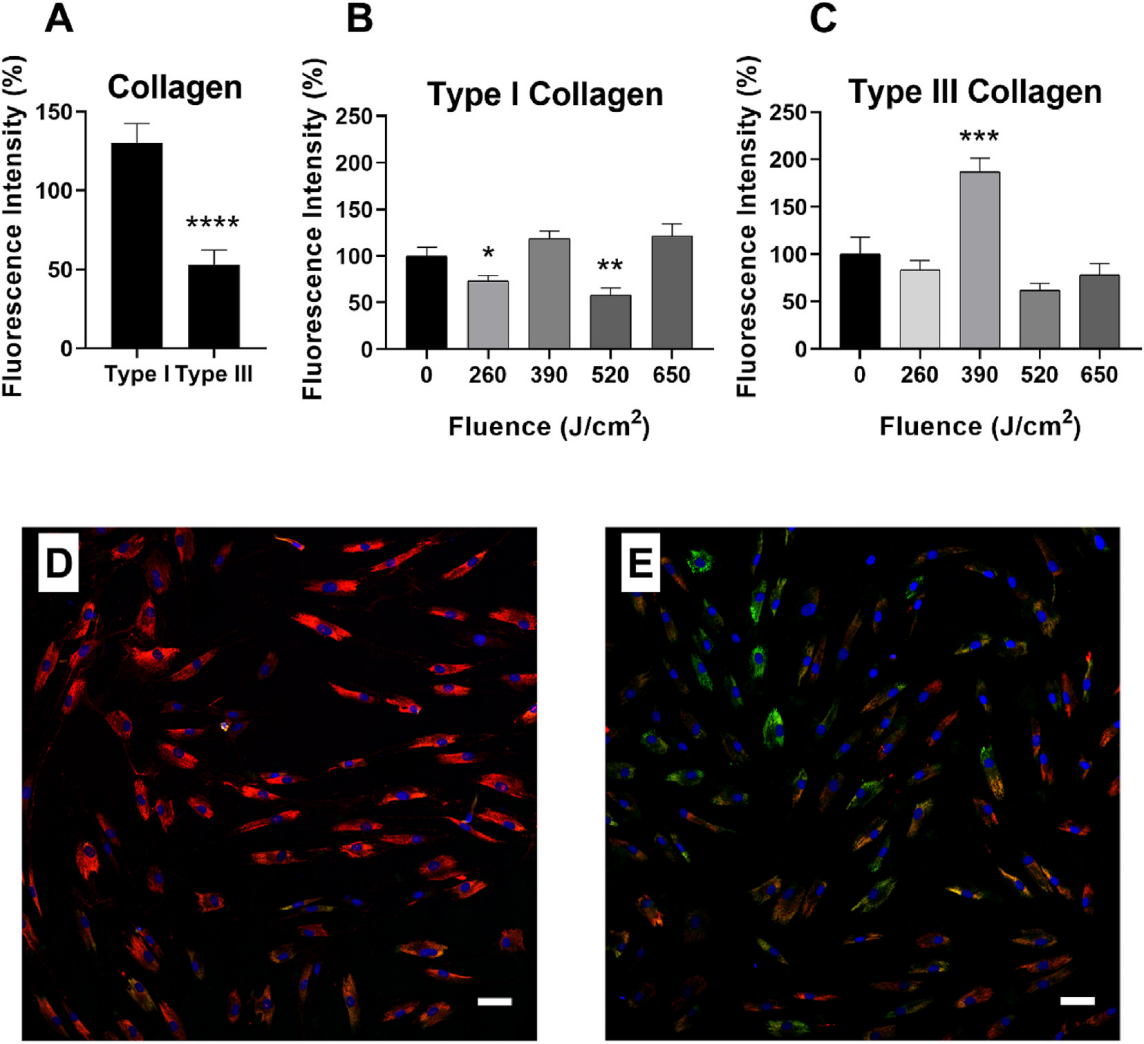
Fluorescence intensity of type I and type III collagen in HDFa control cells, which are not subject to irradiation (A). Fluorescence intensity of type I collagen (B) and type III collagen (C). Data are expressed as the mean ± SEM, n = 20. Statistical analysis: Mann-Whitney test; ∗p < 0.05; ∗∗p < 0.01; ∗∗∗p < 0.001 vs. control cells (unirradiated HDFa cells). The control cells were normalized for the average value of the respective group. Representative images of confocal microscopy of untreated (D) and treated sample (E). Scale bar 50 μm.

First, we measured the fluorescence intensity of type I and III collagen basal expression in HDFa cells that had not been irradiated (Fig. 2A), showing that basal expression of type III collagen is significantly lower than the expression of type I collagen. In Fig. 2B, the application of 260 and 520 J/cm^2^ induces a significant reduction of fluorescence intensity of type I collagen. Only a dose of 390 J/cm^2^ elicits a significant increase in the expression of type III collagen (Fig. 2C). Fig. 2D and E show a representative confocal acquisition of unirradiated (D) and treated (E) samples.

## 4 Discussion

In healthy human skin, types I and III collagen are expressed ranging from 80 to 85% and 10–15%, respectively [18]. Currently, at least fourteen different types of collagen are known, most of which are in the connective tissue. Among them, the major part plays a crucial role during the formation of the skin, and their content fluctuations and relationship have been observed during ageing. In particular, type I collagen makes an essential contribution to the skin concerning thickness, whereas type III collagen is more involved in forming the skin's reticular structure [19]. In terms of morphology, a negative relationship was discovered between fiber diameter and the type I/III collagen content ratio. Therefore, morphological differences between scarred and normal skin are likely due to the exuberant accumulation of type I collagen and the consequent change in the balance of type I/III [19]. It has also been observed that normal skin shows age-dependent differences in total collagen content, which decreases with age, mainly the type III collagen, leading to a progressively increased type I/III collagen ratio. This factor may be associated with a prevalence of permanent scar tissue. The age-dependent increase in the relationship of type I/III content is above one for adolescent, while type I and III collagen content are lower in the elderly age group [19]. These data about age-dependent levels of collagen types and skin recovery can provide primary prevention of skin health in rejuvenation and hypertrophic scar formation.

In numerous wound models, laser wavelengths ranging from 524 to 904 nm have been shown to accelerate wound healing, boost collagen formation, promote epithelial differentiation, and stimulate dermal vascularity [20]. Additionally, the 636 nm laser ability to promote cell proliferation and promote wound healing in fibroblasts can control oxidative stress [21].

In rat model, the use of 685 nm applying a dose of 20 J/cm^2^ stimulates collagen deposition, increases myofibroblasts cells, and improves the reorganization of healed tissue [22].

Several studies conducted on cultured fibroblasts reveal that 812 nm increases DNA production, 860 nm promotes cellular proliferation, 660 nm up-regulates basic fibroblastic growth factor release and 632.8 nm induces the activation of fibroblasts into myofibroblasts [23]. Moore [24] showed that 665 and 675 nm stimulate a faster proliferation in fibroblasts compared to endothelial cells, whereas 810 nm light inhibits this process. These different findings might be caused by a variety of variables, such as the laser irradiation parameters (e.g. wavelength, power density, and fluence), the type of cells exposed to the laser, or an underlying wound healing flaw in *in vivo* systems.

On this basis, the results obtained from our experiments proved to agree with the literature data.

The doses of irradiation used in this work were already successfully applied in the dermatological and aesthetic fields. In particular, these doses are used to reshape and reduce wrinkles and skin scars [[10], [11], [12], [13], [14]]. Our results showed that no dose tested affects fibroblast proliferation and viability. The lack of these effects in our in vitro model suggests that the tested-doses should not affect this parameter directly (e.g. modulating the genes expression or protein synthesis). However, in patients subjected to form hypertrophic scars is possible that the 675 nm irradiation can modulate one or more pathogenic intra- or intercellular signalling pathways, restoring the physiological function. However, to support this hypothesis, long-term patient monitoring studies are needed.

The application of 260 and 520 J/cm^2^ causes a significant decrease in the fluorescence intensity of type I collagen, demonstrating that the collagen I/III ratio can be kept low for always young skin.

Low fluences are not enough to stimulate fibroblasts, while high fluences overcome the collagen denaturation threshold and therefore another tissue remodeling action mechanism can be induced.

These findings on the reorganization of types I and III collagen following laser therapy showed a considerable improvement in type III collagen expression, which suggests laser-induced neocollagenesis activation. We also demonstrated that the application of 390 J/cm^2^ increases the synthesis of type III collagen in treated HDFa, as compared to untreated cells. As already discussed, the same dose does not stimulate an increase in the proliferation rate, thus it can be considered a direct effect on collagen synthesis. We can assume that 675 nm-irradiation directly affects collagen synthesis stimulating neocollagenesis, thus increasing skin elasticity. In addition, the synthesis of type III collagen leads to a better scar outcome in subjects susceptible to the formation of hypertrophic scars, improving the functionality and aesthetic result.

## 5 Conclusion

Our findings demonstrated that the application of 390 J/cm^2^ of 675 nm laser wavelength did not affect cell viability and proliferation, while stimulates type III collagen synthesis in human cultured fibroblasts, confirming its anti-ageing effect in aesthetic field.

Since 2013, when Ken Arndt coined the term “prejuvenation” as well as “to prevent the loss of youth”, the attitude toward aesthetic medicine is changing, and also younger patients are looking for high-performance procedures to maintain their youth and avoid the appearance of the time signs [9]. Although it is obvious that ageing is a complex process that includes persistent sun exposure, elastic fiber deterioration, and cumulative mechanical stress from face muscle contractions [22], on the basis of our results we could consider treatment with 675-nm radiation for the purpose of prejuvenation. The lack of cytotoxic effects, demonstrated by proliferation and viability studies, and the stimulating effect of the dose of 390 J/cm^2^ on the synthesis of type III collagen, suggest that the treatment with 675 nm-radiation can be safe even on young skin, which still does not show age-related signs of ageing. In particular, could be utilized even in younger subjects who are more exposed or genetically predisposed to photoaging [25] and to all those factors known to induce and stimulate skin ageing.

## Author contributions

G.M., F.M., T.Z. and F.R. performed the research and contributed substantially to the study design, interpretation, and data acquisition/analysis; I.F., L.P., and G.M. contributed to the manuscript writing. All authors were involved in the drafting and revision of the manuscript and given final approval of the version to be published. Each author has agreed to be responsible for all aspects of the job to ensure that issues relating to the accuracy or integrity of any part of the job are properly investigated and resolved.

## Funding

Not applicable.

## Declaration of competing interest

L.P., I.F., F.M., and T.Z., are employed at El. En. Group. G.M., and F.R. declare that the research was conducted in the absence of any commercial or financial relationships that could be construed as a potential conflict of interest.

## Data Availability

The data that support the findings of this study are available from the corresponding author upon reasonable request.
